# Joint inference of cell lineage and mitochondrial evolution from single-cell sequencing data

**DOI:** 10.1093/bioinformatics/btae231

**Published:** 2024-06-28

**Authors:** Palash Sashittal, Viola Chen, Amey Pasarkar, Benjamin J Raphael

**Affiliations:** Department of Computer Science, Princeton University, Princeton, NJ 08540, United States; Department of Computer Science, Princeton University, Princeton, NJ 08540, United States; Department of Computer Science, Princeton University, Princeton, NJ 08540, United States; Department of Computer Science, Princeton University, Princeton, NJ 08540, United States

## Abstract

**Motivation:**

Eukaryotic cells contain organelles called mitochondria that have their own genome. Most cells contain thousands of mitochondria which replicate, even in nondividing cells, by means of a relatively error-prone process resulting in somatic mutations in their genome. Because of the higher mutation rate compared to the nuclear genome, mitochondrial mutations have been used to track cellular lineage, particularly using single-cell sequencing that measures mitochondrial mutations in individual cells. However, existing methods to infer the cell lineage tree from mitochondrial mutations do not model “heteroplasmy,” which is the presence of multiple mitochondrial clones with distinct sets of mutations in an individual cell. Single-cell sequencing data thus provide a mixture of the mitochondrial clones in individual cells, with the ancestral relationships between these clones described by a mitochondrial clone tree. While deconvolution of somatic mutations from a mixture of evolutionarily related genomes has been extensively studied in the context of bulk sequencing of cancer tumor samples, the problem of mitochondrial deconvolution has the additional constraint that the mitochondrial clone tree must be concordant with the cell lineage tree.

**Results:**

We formalize the problem of inferring a concordant pair of a mitochondrial clone tree and a cell lineage tree from single-cell sequencing data as the Nested Perfect Phylogeny Mixture (NPPM) problem. We derive a combinatorial characterization of the solutions to the NPPM problem, and formulate an algorithm, MERLIN, to solve this problem exactly using a mixed integer linear program. We show on simulated data that MERLIN outperforms existing methods that do not model mitochondrial heteroplasmy nor the concordance between the mitochondrial clone tree and the cell lineage tree. We use MERLIN to analyze single-cell whole-genome sequencing data of 5220 cells of a gastric cancer cell line and show that MERLIN infers a more biologically plausible cell lineage tree and mitochondrial clone tree compared to existing methods.

**Availability and implementation:**

https://github.com/raphael-group/MERLIN.

## 1 Introduction

Human cells, like nearly all eukaryotic cells, contain organelles called mitochondria that replicate independently from the nuclear genome. The number of mitochondria in a cell ranges from 100 to 1000 s depending on the cell type. The mitochondria in a cell continue to replicate and die, even when the cell is not dividing. During the replication process, somatic mutations may occur in the mitochondrial genomes, also known as mitochondrial DNA (mtDNA), and the fraction of genomes with a certain mutation, known as the “heteroplasmy,” varies over time (Chinnery and Samuels 1999, Stewart and Chinnery 2015, Aryaman *et al.* 2018, Lawless *et al.* 2020). When a cell divides, its mitochondria are distributed roughly equally (Mishra and Chan 2014) between the two daughter cells, and thus, mitochondrial mutations are heritable.

In recent years, there has been increasing recognition of the utility of mitochondrial mutations, which were traditionally overlooked in single-cell analyses, to trace the lineage of individual cells (Ludwig *et al.* 2019, Xu *et al.* 2019, Lareau *et al.* 2021, Velten *et al.* 2021, Lareau *et al.* 2022, Penter *et al.* 2023). Notably, there are several advantages of using mitochondrial mutations over nuclear mutations to infer the cell lineage tree. The relatively higher mutation rate of the mitochondrial genome compared to the nuclear genome (∼10-fold) (Xia 2012, Stewart and Chinnery 2015) enables inference of cell lineage trees with higher resolution. Moreover, mitochondrial mutations can be measured with several single-cell sequencing technologies, including single-cell RNA sequencing (Ludwig *et al.* 2019, Miller *et al.* 2022), single-cell ATAC (Lareau *et al.* 2021, 2023a) and single-cell DNA sequencing (Andor *et al.* 2020), that concurrently measure diverse characteristics of the cell. As such, accurate lineage tracing from such data would provide crucial information about the changes in cell state during biological processes. Lastly, mitochondrial mutations occur naturally allowing retrospective lineage tracing of cells unlike recently developed dynamic lineage tracing technologies (McKenna *et al.* 2016, Kalhor *et al.* 2018, Raj *et al.* 2018, Wagner *et al.* 2018, Chan *et al.* 2019, Gong *et al.* 2022, Yang *et al.* 2022) that can only be employed on engineered cell lines and model organisms.

Presently, there are no methods specifically designed for lineage tracing using mitochondrial mutations. While several studies (Ludwig *et al.* 2019, Lareau *et al.* 2021, Velten *et al.* 2021, Kwok *et al.* 2022, 2023b, Penter *et al.* 2023) have demonstrated the utility of mitochondrial lineage tracing to study developmental systems, these studies either employ traditional distance-based phylogeny inference methods (Michener and Sokal 1957, Saitou and Nei 1987) or specialized methods for single-cell DNA sequencing of nuclear mutations (Jahn *et al.* 2016, Malikic *et al.* 2019a) to build the cell lineage trees. Importantly, these methods do not model two important characteristics that are unique to mitochondrial mutations. First, while each cell harbors a single nuclear genome, it may contain multiple “mitochondrial clones,” i.e. subpopulation of mitochondrial genomes with different sets of somatic mutations. Second, all these mitochondrial clones found across different cells are evolutionarily related to each other by the “mitochondrial clone tree,” where nodes are mitochondrial clones and edges are mutations that distinguish different mitochondrial clones. Using methods that do not model these features of mitochondrial mutations could result in inaccurate inference of the cell lineage tree.

A key challenge in using mitochondrial mutations for lineage tracing is that, while single-cell sequencing measures the single nuclear genome, it yields a superposition of all mitochondrial clones in the cell. Specifically, for each cell, we get sequencing data from a mixture of mitochondrial clones in that cell. Recovering the mitochondrial clones and their evolution history, i.e. the mitochondrial clone tree, requires deconvolution of mixed mitochondrial data obtained from single-cell sequencing. These data are similar to the mixed data generated by bulk sequencing of cancer tumors (Nik-Zainal *et al.* 2012) that comprise subpopulation of cells with distinct sets of mutations called “cancer clones.” Several methods have been developed to deconvolve bulk data from multiple tumor samples to infer the cancer clones and their evolutionary history (Deshwar *et al.* 2014, Zare *et al.* 2014, Malikic *et al.* 2015, 2019b, Popic *et al.* 2015, Jiang *et al.* 2016, Salcedo *et al.* 2020, Wintersinger *et al.* 2022). At first glance, it might seem these methods can be directly applied to single-cell mitochondrial data to reconstruct the mitochondrial clone tree. However, these methods do not model a unique feature of mitochondrial evolution—the mitochondrial clone tree must be “concordant” with the cell lineage tree of the sequenced cells. Specifically, for a mitochondrial clone to arise in a cell, its ancestral mitochondrial clone must already be present in that cell. In contrast, there is no such constraint in the bulk-sequencing deconvolution problem encountered in cancer phylogenetics.

We formalize the problem of finding a concordant pair of a cell lineage tree and a mitochondrial clone tree from single-cell sequencing data. Specifically, we pose the problem of deconvolving the mitochondrial mutational frequencies derived from single-cell sequencing data as a “structured matrix factorization problem.” Importantly, not all pairs of cell lineage trees and mitochondrial clone trees are concordant. We introduce the concept of “nested perfect phylogenies” that characterizes the combinatorial structure of concordant pairs of cell lineage trees and mitochondrial clone trees under the “perfect phylogeny model” (Gusfield 1991). Using this characterization, we develop MERLIN (Mitochondrial EvolutionaRy Lineage INference), the first algorithm to simultaneously trace the lineage of cells and reconstruct the evolutionary history of mitochondrial clones from single-cell sequencing data.

On simulated data, we show that MERLIN outperforms both single-cell lineage tracing methods tailored for nuclear mutations, and cancer clone tree inference methods designed for bulk-sequencing data. On single-cell whole-genome sequencing data of 5220 cells of a gastric cancer cell line (MKN-45) (Andor *et al.* 2020), MERLIN infers a cell lineage tree that is consistent with copy number variation analysis of the data from the original study. Moreover, MERLIN also infers a concordant mitochondrial clone tree that reveals the dynamics of mitochondrial evolution in this cell line.

## 2 Cell lineage tree, mitochondrial evolution, and nested perfect phylogenies

Suppose we sequence *n* cells in an experiment and measure *m* mitochondrial mutations (or mutation clusters) across these cells. For each mutation, the single-cell sequencing data indicate the fraction of mitochondrial genomes in each cell that contains that mutation. We represent this data by a *n *×* m* frequency matrix $F=\left[f_{ij}\right]$, where *f_ij_* is the frequency of mutation *j* in cell *i*. Our goal is to infer the evolutionary history of the mitochondria and the ancestral relationship between the cells using the frequency matrix *F*.

The evolutionary history of the mitochondrial mutations is represented by a mitochondrial clone tree *S*, where the edges represent mitochondrial mutations. Each vertex, other than the root, represents a mitochondrial clone that is characterized by the mutations that occur along the path from the root of *S* to the vertex. We make the infinite sites assumption (Kimura 1969), i.e. we assume that there are no parallel mutations or back mutations during the evolution of the mitochondrial clones. As such, each mutation occurs exactly once in the clone tree. Thus, the clone tree has *m* vertices excluding the root, each of which represents a distinct mitochondrial clone. Such a tree is called a “perfect phylogeny” and can be alternatively described by a “perfect phylogeny matrix” (Gusfield 1991) which indicates the presence/absence of mutations in each of the *m* clones. Specifically, perfect phylogeny matrix $B=\left[b_{\ell ,j}\right]$ for clone tree *S* is a *m *×* m* binary matrix, where $b_{\ell ,j}=1$ if mutation *j* is present in mitochondrial clone ℓ and $b_{\ell ,j}=0$ otherwise.

The single-cell sequencing experiment measures mutations from a mixture of mitochondrial genomes present in unknown proportions in each cell. Let $U=\left[u_{i,\ell }\right]$ be the *n *×* m* “mixture matrix,” where $u_{i,\ell }$ is the proportion of mitochondrial clone ℓ in cell *i*, i.e. $u_{i,\ell }\geq 0$ and $\sum_{\ell =1}^{m}u_{i,\ell }\leq 1$. If there is no bias or sequencing error, then we would expect the fraction of mitochondrial genomes in a cell with a mutation must be the sum of the proportions of mitochondrial clones in the cell with that mutation, i.e. *F*=*UB*.

The problem of factorizing a frequency matrix *F* into a mixture matrix *U* and perfect phylogeny matrix *B*, known as the Perfect Phylogeny Mixture (PPM) problem (El-Kebir *et al.* 2015), has been extensively studied in cancer phylogenetics (Deshwar *et al.* 2014, Zare *et al.* 2014, Malikic *et al.* 2015, Popic *et al.* 2015, Jiang *et al.* 2016, Malikic *et al.* 2019b, Salcedo *et al.* 2020, Wintersinger *et al.* 2022). This problem is used to study the evolution of cancer clones, i.e. subpopulation of cancer cells with distinct sets of mutations, from mixed data generated by bulk sequencing of multiple samples from the same tumor. Specifically, each tumor sample comprises cells from multiple cancer clones in unknown proportions, and bulk sequencing data yield mutational frequencies which indicates the fraction of cells in the sample containing each mutation. The evolution of the cancer clones is modeled by a perfect phylogeny and the PPM problem is formally posed as follows (El-Kebir *et al.* 2015).

Problem 2.1 (Perfect Phylogeny Mixture (PPM)). *Given a frequency matrix F, find a factorization F = UB, if one exists, such that U is a mixture matrix and B is a perfect phylogeny matrix.*

While there are several similarities with the PPM problem, a unique feature of our problem is that, apart from the evolutionary relationships of mitochondrial clones, the sequenced cells are also related to each other by a cell lineage tree. When a cell divides, the mitochondrial genomes in the cell are distributed to the two daughter cells. As such, the proportions of mitochondrial clones in the daughter cells depend on the proportions of the clones in the parent cell. In contrast, in the PPM problem in cancer phylogenetics, while the cancer clones are evolutionarily related, there is no phylogenetic relationship between the tumor samples.

**Figure 1.**
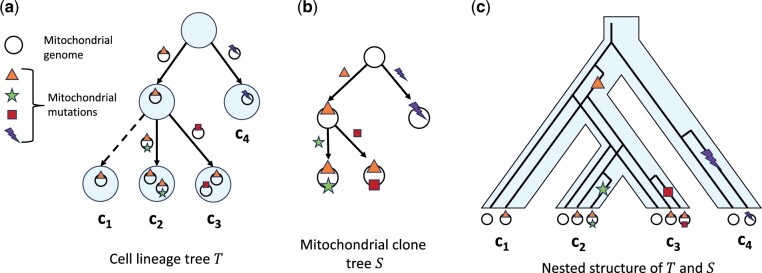
Schematic showing the nested structure of concordant cell lineage and mitochondrial clone trees. (a) The cell lineage tree describing the ancestral relationship of cells. Leaves represent extant cells and internal vertices represent ancestral cells. Edges are annotated by mitochondrial clones depicting the event when a mitochondrial clone arises in a cell due to a somatic mutation. (b) Mitochondrial clone tree describing the evolution of mitochondria. Vertices represent mitochondrial clones and edges are annotated by mitochondrial mutations. (c) Nested structure of the cell lineage tree and the mitochondrial clone tree, where the mitochondrial clone tree is nested within the cell lineage tree.

The phylogenetic relationships between cells can be represented by a cell lineage tree *T*, where the leaves represent the sequenced cells and the internal nodes represent ancestral cells (Fig. 1a). The root of the tree denotes the founder cell that only contains wild-type mitochondrial genomes, i.e. genomes do not have any somatic mutations. The edges of *T* are annotated by mitochondrial clones describing the event when a mitochondrial clone arose in a cell for the first time due to a mitochondrial somatic mutation. Since the mitochondrial mutations follow the infinite sites assumption, it follows that each mitochondrial clone can arise at most once in some cell. Moreover, when a cell divides, the mitochondrial clones in the parent cell are distributed between the two daughter cells. Since mitochondria are distributed roughly equally between the two daughter cells during cell division (Mishra and Chan 2014), we assume that both daughter cells inherit all the mitochondrial clones present in the parent cell at the time of division. We also assume that mitochondrial clones do not go extinct in a nondividing cell, which is a reasonable assumption for clones with high relative abundances. Putting these assumptions together, once a mitochondrial clone arises in a cell, it will be present in all of its descendants. As such, similar to the mitochondrial clone tree, *S* (Fig. 1b), the cell lineage tree is also a perfect phylogeny, where the mitochondrial clones serve as phylogenetic markers, which puts constraints on the structure of the mixture matrix *U*Gusfield (1991). Specifically, the binarization U′ of *U*, where $u\prime_{i,\ell }=1$ if $u_{i,\ell }>0$ and $u\prime_{i,\ell }=0$ if $u_{i,\ell }=0$, must be a perfect phylogeny matrix.

The cell lineage tree and the mitochondrial clone tree for the same biological system must be evolutionary concordant with each other. Specifically, for a mitochondrial clone to arise in a cell, its ancestral mitochondrial clone must already be present in that cell. Since the mitochondria are evolving “within” cells and being inherited by cells during cell division, we can represent the joint process by “nested perfect phylogenies,” where the mitochondrial clone tree is nested “within” the cell lineage tree (Fig. 1).

We formally define the concordance between the cell lineage tree *T* and mitochondrial clone tree *S* by the partial orders induced by the trees on the mitochondrial clones. Let M denote the set of mitochondrial clones and $\left(M,{⪯}_{T}\right)$ be the partial order induced by the cell lineage tree *T* whose edges are annotated by the mitochondrial clones. Similarly, let $\left(M,\;{⪯}_{S}\right)$ be the partial order induced by the mitochondrial clone tree *S* whose vertices represent the mitochondrial clones. For *T* and *S* to be concordant, we require that partial order $\left(M,{⪯}_{T}\right)$ is an “extension” of the partial order $\left(M,\;{⪯}_{S}\right)$; i.e. for any pair (ℓ,ℓ′) of mitochondrial clones, ℓ ⪯S ℓ′ implies ℓ ⪯T ℓ′. We formally define the concordance between a mitochondrial clone tree *S* and a cell lineage tree *T* as follows.

Definition 1. A mitochondrial clone tree *S* and cell lineage tree *T* on a set M of mitochondrial clones are “concordant” if and only if $\left(M,{⪯}_{T}\right)$ is an extension of $\left(M,{⪯}_{S}\right)$.

Further, we say that clone matrix *B* and binarization U′ of mixture matrix *U* are “concordant” if the corresponding mitochondrial clone tree and cell lineage tree are concordant.

The nested structure of the cell lineage tree and the mitochondrial clone tree in the nested PPM is similar to how gene trees are nested (embedded) within species trees in the context of population genetics under the multispecies coalescence (MSC) model (Jiao *et al.* 2021). The MSC process (Jiao *et al.* 2021) models the evolutionary relationship between individual across several species, where each species may comprise multiple alleles/haplotypes of each gene. Interestingly, the evolution of the multiple alleles of a gene across the species is akin to the evolution of mitochondrial clones across cells. Moreover, the frequency of individuals of a species carrying an allele of a gene varies over time, just as the proportion of mitochondrial clones in a cell vary over time. As such, the gene tree in the MSC model corresponds to the mitochondrial clone tree, while the species tree, which describes the evolutionary relationship between the species, corresponds to the cell lineage tree. We elaborate on the implications of this correspondence in Section 6.

**Figure 2.**
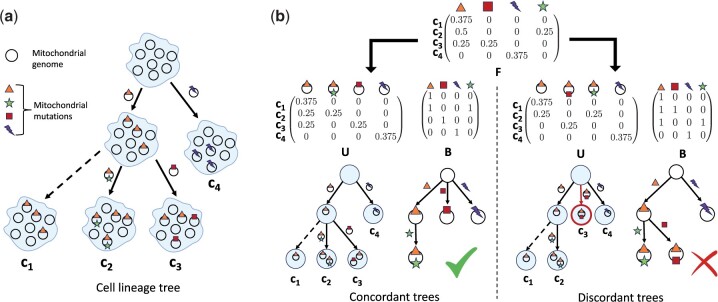
Overview of mitochondrial evolution and the concordance between the cell lineage tree and the mitochondrial clone tree. (a) The cell lineage tree describing the phylogenetic relationships between the cells. Each cell contains multiple mitochondrial genomes (circles) that acquire mutations. Edges are annotated by mitochondrial clones depicting the event when a mitochondrial clone arises in a cell due to a somatic mutation. (b) The measured frequency matrix *F* of the sequenced cells describes the fraction of mutated mitochondrial genomes in each cell. There are generally multiple factorizations *F*=*UB* into a mixture matrix *U* whose entries are the proportions of mitochrondria in each cell—and that corresponds to a cell lineage tree—and a mutation matrix *B* that corresponds to a mitochondrial clone tree. Some factorizations correspond to concordant cell lineage and mitochondrial clone trees (left), while for other factorizations the trees are discordant.

There may be multiple factorizations of a frequency matrix *F* =*UB*, such that both *B* and binarization U′ of the mixture matrix *U* are perfect phylogeny matrices. However, some of these solutions may not be nested perfect phylogenies (Fig. 2b). This motivates the problem where we must find a factorization *F*=*UB*, where *U* supports a perfect phylogeny, *B* is a perfect phylogeny matrix, and *U* and *B* are concordant. We refer to this as the Exact Nested Perfect Phylogeny Mixture (ENPPM) Problem.

Problem 2.2 (Exact Nested Perfect Phylogeny Mixture Problem (ENPPM)). *Given an n × m frequency matrix F, find factorization F = UB such that B and a binarization* U′*of U are perfect phylogeny matrices, and these matrices are concordant.*

In practice, the frequency matrix obtained from sequencing data contain errors and only approximates the true mutational frequencies. We deal with this uncertainty by minimizing the *L*_1_ error $|F-UB{|}_{1}$ where mixture matrix *U* and clone matrix *B* determine the evolutionarily concordant cell lineage tree and mitochondrial clone tree, respectively. We refer to this as the Nested Perfect Phylogeny Mixture (NPPM) Problem.

Problem 2.3 (Nested Perfect Phylogeny Mixture Problem (NPPM)). *Given a frequency matrix F, find mixture matrix U and clone matrix B, such that B and binarization* U′*of U are perfect phylogeny matrices that are concordant, and* $|F-UB{|}_{1}$*is minimized.*

### 2.1 Model assumptions

Here, we explicitly state the assumptions made by the “Nested PPM.” First, we assume, as in previous works on clonal reconstruction (Jiao *et al.* 2014, El-Kebir *et al.* 2015, Jahn *et al.* 2016, Malikic *et al.* 2019a, Sashittal *et al.* 2023), that mutations follow the “infinite sites assumption” (Farris 1977), which states that each mutation can occur at most once during clonal evolution. Second, we assume that mitochondrial clones do not go extinct and, during cell division, both daughter cells inherit all mitochondrial clones present in the parent cell. This is a reasonable assumption for cases where cells have been shown to maintain mitochondrial heteroplasmy (Gupta *et al.* 2023) and when only high-frequency mutations are analyzed, making loss of the clone unlikely during cell division. In Section 6, we describe the implications of relaxing this assumption.

## 3 Characterizing the mixture of nested perfect phylogenies

We derive a characterization of the solutions of the ENPPM problem by building on previous work (El-Kebir *et al.* 2015) on the characterization of solutions of the PPM problem (Problem 0.1). Note that the ENPPM problem is a constrained version of the PPM problem, where we additionally require the binarization U′ of *U* to be a perfect phylogeny matrix that is concordant with *B*.

Underpinning the characterization of solutions to the PPM problem is the concept of an ancestry graph *G*. For a frequency matrix *F*, *G* is a directed graph has *m *+* *1 vertices {0,1,…,m} and edge (j,j′) exists if and only if either *j *=* *0 or $f_{i,j}\geq f_{i,j\prime }$ for each row *i* in the frequency matrix. Let $\delta_{G}^{+}(j)$ denote the set of target vertices of the outgoing edges at vertex *j* in *G*. El-Kebir *et al.* (2015) proved that a frequency matrix admits a solution the PPM problem if and only if the ancestry graph contains a spanning arborescence that satisfies the “sum condition” defined in the following theorem.

Theorem 1. *A frequency matrix F admits a solution to the PPM problem if and only if there exists a spanning arborescence H of ancestry graph G of F satisfies the sum condition, i.e.*

$$f_{i,j}\geq \sum_{j\prime \in \delta_{H}^{+}(j)}f_{i,j\prime },$$

*for all* i∈{1,…,n}.

Importantly, the spanning arborescences in the ancestry graph correspond to candidate clone trees. Specifically, we obtain a clone tree *S* by annotating the incoming edge of each vertex *j* in a spanning arborescence by mutation *j*. Vertex 0 in the obtained graph represents the wildtype mitochondria and vertices {1,…,m} represent the *m* mitochondrial clones. The spanning arborescence *H* that satisfies the “sum condition” determines the clone tree *S*, and consequently the clone matrix *B*, in the solution to the PPM problem.

While the PPM problem only requires *U* to be a mixture matrix in the factorization *F*=*UB*, the ENPPM problem additionally requires the binarization U′ of *U* to be a perfect phylogeny matrix. We show that this translates to the requirement that the binarization F′ of the frequency matrix *F* is a perfect phylogeny matrix. Further, we prove that for the mixture matrix *U* and clone matrix *B* to be concordant (Definition 1), the frequency $f_{i,j}$ must be strictly greater than $\sum_{j\prime \in \delta_{H}^{+}(j)}f_{i,j\prime }$ for some spanning arborescence *H* of *G* if $f_{i,j}>0$, and $\sum_{j\prime \in \delta_{H}^{+}(j)}f_{i,j\prime }=0$ otherwise. Putting these conditions together we derive the following characterization of frequency matrices that admit a solution to the ENPPM problem.

Theorem 2. *A frequency matrix F admits a solution to the ENPPM problem if and only if (i) binarization* F′*of F is a perfect phylogeny matrix and (ii) there exists a spanning arborescence H of ancestry graph G of F such that, for all* $i\in \{1,\ldots ,n\},\;f_{i,j}>\sum_{j\prime \in \delta_{H}^{+}(j)}f_{i,j\prime }$*if* $f_{i,j}>0$*and* $\sum_{j\prime \in \delta_{H}^{+}(j)}f_{i,j\prime }=0$*if* $f_{i,j}=0$.

While checking if a matrix is a perfect phylogeny, which is condition (i) in Theorem 2, can be performed in polynomial time (Gusfield 1991), we show that checking if a frequency matrix admits a solution to the ENPPM problem is NP-hard using a reduction from the Subset Sum Problem (Lewis 1983).

Theorem 3. *The ENPPM problem is NP-complete, even when n = 2.*

We provide proofs for the theorems stated above in Supplementary Section SA.

## 4 MERLIN: a mixed integer linear program for the NPPM problem

Here, we present MERLIN (Mitochondrial evolutionaRy Lineage INference), an algorithm that uses a mixed integer linear program (MILP) to solve Problem 2.3 and Problem 2.2. We start by building an ancestry graph *G* such that the mitochondrial clone tree *S* is determined by a spanning arborescence of *G*. For Problem 2.2, where the frequency matrix has no errors, the ancestry graph *G* is constructed using the frequency matrix as described in the previous section. For Problem 2.3, we must account for noise in the frequency matrix. We describe the more robust procedure to construct an ancestry graph *G* in Supplementary Section SB.1. To promote sparsity and avoid overfitting the noisy frequency data, we require that if a mitochondrial clone is present in a cell, it must have a minimum abundance of *μ*. For a given frequency matrix *F* and the ancestry graph *G*, the MILP finds a mixture matrix *U* and clone matrix *B* that are concordant, such that $|F-UB{|}_{1}$ is minimized.

The MILP is based on the characterization of concordant mixture and clone matrices described in the previous section. The MILP comprises constraints for (i) enforcing concordance between the clone tree and cell lineage tree and (ii) enforcing that the binarization of the mixture matrix admits a perfect phylogeny. We describe the constraints for (i) here and refer to Supplementary Section SB.2 for (ii).

### 4.1 Clone tree

We introduce a binary variable *x_e_* for each edge *e* in the ancestry graph *G* to indicate if the edge is present in the clone tree *S*, which as described in the previous section, is determined by the clone matrix *B*. Since *S* is a spanning arborescence of the ancestry graph *G*, exactly one of the incoming edges of each vertex corresponding to a mitochondrial mutation must be present in *S*. We encode this by enforcing the following constraints.

$$\sum_{e\in \delta_{G}^{-}(j)}x_{e}=1,\text{for all}\;j\in \{1,\ldots ,m\},$$

where $\delta_{G}^{-}(j)$ is the set of incoming edges to vertex *j* in ancestry graph *G*.

### 4.2 Mixture matrix

We introduce continuous variables $u_{i,\ell }$ for each cell *i* and clone ℓ to represent the mixture matrix *U*. We also introduce binary variables $u\prime_{i,\ell }$ to represent the binarization of each entry $u_{i,\ell }$ in the mixture matrix *U*. Since U′ is the binarization of *U*, we enforce the following constraints for each cell *i* and clone ℓ.

$$u_{i,\ell }\leq u\prime_{i,\ell }\;\;\text{and}\;\;u_{i,\ell }\geq \mu u\prime_{i,\ell },$$

where *μ* is the minimum value of proportion of a mitochondrial clone present in a cell. Further, since *U* is a mixture matrix, we require the following constraint for each cell *i*.

$$\sum_{\ell =1}^{m}u_{i,\ell }\leq 1.$$

### 4.3 Concordance

To ensure concordance between the clone tree *S* and the mixture matrix *U* we require that the partial order ${⪯}_{T}$ induced by the cell lineage tree *T* corresponding to the *U* is an extension of the partial order ${⪯}_{S}$ induced by *S*. As such, we require that for an edge (ℓ,ℓ′) in *S* indicating ℓ⪯S ℓ′, if cell *i* contains clone ℓ′, then cell *i* should also contain clone ℓ since ℓ⪯T ℓ′. We encode this by the following constraint for each edge e=(ℓ,ℓ′) in *G* and cell *i*.

$$u\prime_{i,\ell }\geq x_{e}+u\prime_{i,\ell \prime }-1.$$

### 4.4 Frequency matrix

We introduce continuous variables ${\hat{f}}_{i,j}$ for each cell *i* and mutation *j* to represent the entries of the matrix $\hat{F}=UB$. Recall that the frequency ${\hat{f}}_{i,j}$ of a mutation *j* in cell *i* is given in El-Kebir *et al.* (2015) by:

$${\hat{f}}_{i,j}=u_{i,j}+\sum_{\left(j,j\prime )\in \delta_{S}^{+}(j\right)}{\hat{f}}_{i,j\prime }.$$

We incorporate the contribution of each edge of *G* to the frequency of a mutation in a cell by introducing continuous variables $h_{i,e}$ that encode the product $x_{e}{\hat{f}}_{i,e}$ using the following constraints for each cell *i* and edge e=(j,j′) in *G*:

$$\begin{array}{c} h_{i,e}\leq {\hat{f}}_{i,j\prime },\\ h_{i,e}\leq x_{e},\\ h_{i,e}\geq {\hat{f}}_{i,j\prime }+x_{e}-1. \end{array}$$

Using $h_{i,e}$, we enforce the following constraints for each cell *i* and mutation *j* to compute ${\hat{f}}_{i,j}$.

$${\hat{f}}_{i,j}=u_{i,j}+\sum_{e\in \delta_{G}^{+}(j)}h_{i,e}.$$

### 4.5 Objective function

We encode the *L*_1_ norm of the error *F* and $\hat{F}=UB$ by introducing continuous variables $c_{i,j}\in [0,1]$ for each cell *i* and mutation *j* and imposing the constraints,

$$c_{i,j}\geq f_{i,j}-{\hat{f}}_{i,j},\;c_{i,j}\geq {\hat{f}}_{i,j}-f_{i,j}.$$

In order to minimize the $|F-\hat{F}{|}_{1}$ we set minimize the objective function $\sum_{i=1}^{n}\sum_{j=1}^{m}c_{i,j}$. This MILP has $O(m^{2}+nm)$ binary variables, *O*(*nm*) continuous variables, and *O*(*nm*) constraints. In cases with multiple valid solutions to this optimization problem, MERLIN returns the first optimal solution it finds.

## 5 Results

### 5.1 Simulated data

We compare the performance of MERLIN against existing methods on simulated data. We generated simulated data with *n *=* *50, 100, 500 cells and *m *=* *5, 10, 15 mitochondrial mutations. We used a growing random network (Krapivsky and Redner 2001) to generate a clone tree representing the ancestral relationships among mitochondrial clones, and assigned mutations to each clone. The cell lineage tree for *n* cells was generated in such a way that it is concordant with the simulated mitochondrial clone tree (details in Supplementary Section SC). We simulate the sequencing data for each mutation in each cell using a beta-binomial read count model (details in Supplementary Section SC.3). We simulate five instances for each combination of the varying simulation parameters.

Since there are no methods specifically designed for mitochondrial clone tree reconstruction from single-cell sequencing data, we consider two classes of methods for comparison. Firstly, we compare the accuracy of cell lineage tree inferred by MERLIN against three single-cell phylogeny inference methods, SCITE (Jahn *et al.* 2016), PhISCS (Malikic *et al.* 2019a) and PhISCS-BnB (Sadeqi Azer *et al.* 2020), designed for single-cell sequencing data with nuclear mutations. These methods generate a “mutation matrix,” i.e. a cell-by-mutation binary matrix that indicates the presence/absence of mutations in each cell, which determines the cell lineage tree. The input of these methods comprises an “observed mutation matrix” derived from the simulated frequency matrix which indicates putative presence/absence of mitochondrial mutations in each cell along with estimated false-positive mutation rate *α* and false-negative mutation rate *β*. Note that the false-positive mutation rate and false-negative mutation rate here may differ from that in cancer cells. Supplementary Section SC describes the procedure to generate these inputs from the simulated data. Secondly, we compare the performance of MERLIN against clone tree inference methods, such as AncesTree (El-Kebir *et al.* 2015), CALDER (Myers *et al.* 2019), Pairtree (Wintersinger *et al.* 2022), designed to deconvolve multi-sample bulk sequencing data of cancer tumors. These methods take the variant and total read counts for each mitochondrial mutation in each cell as input and generate a cell-by-clone mixture matrix and a mitochondrial clone tree. Since AncesTree (El-Kebir *et al.* 2015), failed to produce a mitochondrial clone tree *S* in more than 70% of the simulation instances, we only compare the performance of MERLIN against CALDER and Pairtree. We note that CALDER uses the same model as Ancestree when there is no temporal ordering of the samples, and therefore the outputs are expected to be identical. Supplementary Section SD describes the precise input parameters used for each method in this study.

We compare the performance of the competing methods using two metrics. First, we compare the accuracy of the methods to infer the cell lineage by comparing the mutational profile of the cells with the ground-truth. Specifically, following previous studies (El-Kebir 2018, Sashittal *et al.* 2023), we compute the “mutation matrix error,” which is the fraction of entries in the mutation matrix inferred by the methods that do not match the ground–truth mutation matrix. The mutation matrix error is 0 if and only if the inferred mutation matrix matches the ground-truth exactly. For MERLIN and Pairtree, the mutation matrix is given by the binarization of the factorized $\hat{F}=UB$ matrix. We evaluate the accuracy of the mitochondrial clone tree, through the “parent–child distance” $D(S,S^{*})$ (Govek *et al.* 2018, Aguse *et al.* 2019, Sashittal and El-Kebir 2020), which is the size of the symmetric difference of the edges sets of the inferred mitochondrial clone tree *S* and the true clone tree $S^{*}$, normalized by the number *m* of mutations. The parent–child distance is a metric in the space of clone trees (Govek *et al.* 2018) and, as such, $D(S,S^{*})$ is 0 if and only if the two clone trees *S* and $S^{*}$ are identical.

**Figure 3.**
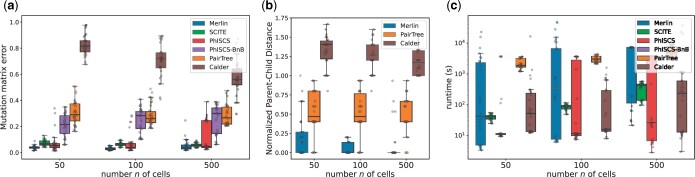
MERLIN outperforms existing methods in reconstructing the mitochondrial clone tree and the cell lineage tree on simulated data. (a) Mutation matrix error, (b) normalized parent–child distance, and (c) runtime (in seconds) for each method on the simulated instances. Box plots show the median and the interquartile range (IQR), and the whiskers denote the lowest and highest values within 1.5 times the IQR from the first and third quartiles, respectively.

MERLIN outperforms existing methods both in terms of the mutation matrix error and the mitochondrial clone tree accuracy across all simulated instances while having comparable running time (Fig. 3). For instance, for the largest instance with *n *=* *500 cells, MERLIN achieves the lowest mutation matrix error (median 0.0352) and the lowest normalized parent–child distance (median 0) compared to Pairtree (0.265 and 0.8), CALDER (0.557 and 1.167), SCITE (0.0544 and N/A), PhISCS (0.046 and N/A) and PhISCS-BnB (0.297 and N/A). We believe MERLIN outperforms the competing methods because it models both the mitochondrial heteroplasmy and the concordance of the mitochondrial clone tree with the cell lineage tree. In contrast, single-cell tree inference methods, such as SCITE, PhISCS, and PhISCS-BnB, are designed for nuclear mutations and thus do not model unique characteristics of mitochondrial evolution. Specifically, these methods do not model the presence of evolutionary-related mitochondrial clones in varying proportions in the cells and instead treat each mutation as an independent character. On the other hand, while mixture deconvolution methods for clone tree inference, such as CALDER and Pairtree (Myers *et al.* 2019, Wintersinger *et al.* 2022), account for mitochondrial heteroplasmy, they ignore the concordance between the mitochondrial clone tree and cell lineage tree.

Lastly, we investigated the impact of violations of the nested PPM on the performance of MERLIN on simulated data. Specifically, we performed simulations in which each mutation in a cell is lost with a probability of 0.05 (details in Supplementary Section SE). As such, in these simulations each cell does not inherit all the mitochondrial clones in their parent cells during cell division. MERLIN still performs well on these simulations and outperforms existing methods (Supplementary Section SE), showing its robustness to minor violations of the model assumptions.

### 5.2 scDNA-seq data of gastric cancer cell line

**Figure 4.**
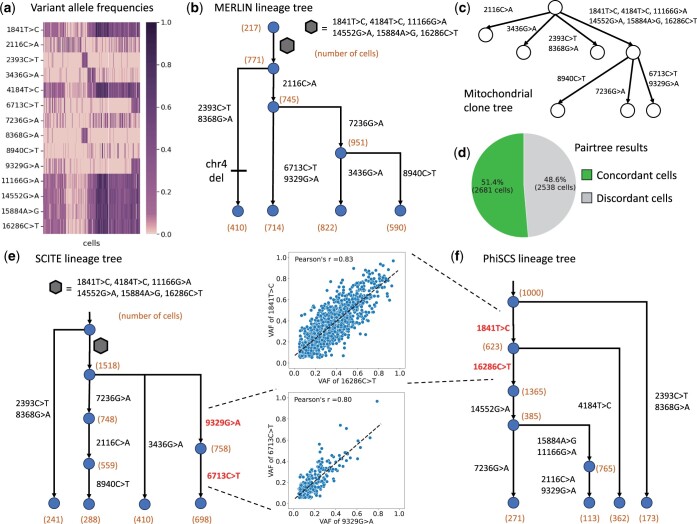
Reconstruction of mitochondrial clone tree and cell lineage tree from single-cell DNA sequencing data. (a) Variant allele frequency matrix for 14 mitochondrial mutations in 5220 cells given as input to MERLIN. (b) The cell lineage tree inferred by MERLIN describing the phylogenetic relationship of the cells with edges annotated by mitochondrial clones. (c) Mitochondrial clone tree inferred by MERLIN describing the evolution of the mitochondrial clones. (d) Proportion of cells in the largest possible cell lineage tree from the Pairtree results that is concordant with the mitochondrial clone tree. Cell lineage tree inferred by (e) SCITE and (f) PhISCS postulate the unlikely occurrence of mutations with highly correlated mutations in separate branches of the trees.

We use MERLIN to analyze a single-cell whole-genome sequencing (scDNA-seq) dataset of 5220 cells from a gastric cancer cell line (MKN-45) with an ultra-low coverage (median 0.022×) of the nuclear genome (Andor *et al.* 2020). Copy number analysis of this data identified only two copy number clones separated by the deletion of the q-arm of chromosome 4. Despite the low coverage of the nuclear genome, which makes identification of nuclear single nucleotide variants in individual cells challenging (Myers *et al.* 2020, Zaccaria and Raphael 2021), the scDNA-seq data contain an average of 8020 mitochondrial reads per cell (Kwok *et al.* 2022). We used MQuad (Kwok *et al.* 2022) to identify 14 mitochondrial mutations across the cells (Fig. 4a). We build an ancestry graph *G* (details in Supplementary Section SB.1) and generate a frequency matrix *F* for the MQuad mutations to feed as input to MERLIN. We compare the results generated by MERLIN with results from SCITE (Jahn *et al.* 2016) and PhISCS (Sadeqi Azer *et al.* 2020), and Pairtree (Wintersinger *et al.* 2022).

MERLIN infers a cell lineage tree that is consistent with the copy number clones in the cell line and the correlations in the mutational frequencies. Specifically, MERLIN clusters the 14 mitochondrial mutations into eight mitochondrial clones, leading to the inference of a lineage tree with seven mutation events that show enrichment of cells with a deletion of the q-arm of chromosome 4 in one of its branches (78% overlap, Fig. 4b). As such, mitochondrial mutations provide a finer resolution of the clones in the sample, compared to nuclear copy number aberrations that only provided two clones.

Unlike MERLIN, SCITE and PhISCS do not model the inheritance of mitochondrial clones between the cells and instead treat each mitochondrial mutation as an independent phylogenetic marker. This leads to the unlikely inference of mutations with highly correlated frequencies across cells occurring in distinct branches of the cell lineage tree inferred by SCITE (6713C >T and 9329G >A, Pearson’s *r *=* *0.83) and PhISCS (1841T >C and 16286C >T, Pearson’s *r *=* *0.8).

MERLIN also produces a mitochondrial clone tree that is concordant with the cell lineage tree and reveals the evolutionary dynamics of the mitochondrial clones (Fig. 4c). The mitochondrial clone tree describes the evolution of eight mitochondrial clones and contains a polytomy (four branches) at the root, with one of the branches annotated by six mutations (1841T >C, 4184T >C, 11166G >A, 14552G >A, 15894A >G, 16286C >T). This mitochondrial clone with six mutations subsequently gives rise to three more clones indicating higher mutability compared to the other clones that do not have any descendants in the clone tree. Importantly, the cell lineage tree produced by MERLIN is concordant with the subclonal structure of the mitochondrial clone tree. In contrast, while Pairtree (Wintersinger *et al.* 2022) produces an identical mitochondrial clone tree, the Pairtree mixture matrix is not concordant with the clone tree. To quantify the discordance in Pairtree results, we compute the minimum number of cells that must be removed from the mixture matrix to yield a cell lineage tree that is concordant with the mitochondrial clone tree (details in Supplementary Section SF). We find that nearly half (2538 out of 5220 cells) of the cells must be removed for Pairtree to generate an evolutionary concordant mixture matrix (Fig. 4d). In summary, MERLIN infers a more biologically plausible mitochondrial clone tree and cell lineage tree from single-cell DNA sequencing data compared to existing methods.

## 6 Discussion

Mitochondria in cells are being continuously destroyed and replicating, acquiring somatic mutations in the process. This results in each cell containing multiple evolutionarily related mitochondrial clones, i.e. subpopulations of mitochondrial genomes with distinct sets of mutations. Several recent studies have shown the utility of these mitochondrial mutations, derived from single-cell sequencing data, in tracing the lineage of cells (Ludwig *et al.* 2019, Xu *et al.* 2019, Lareau *et al.* 2021, Velten *et al.* 2021, Lareau *et al.* 2022, Penter *et al.* 2023). However, all these lineage tracing studies have employed computational tools designed for nuclear mutations that do not model mitochondrial evolution and the presence of multiple mitochondrial clones (heteroplasmy) in the same cell. Here, we introduce MERLIN, a new algorithm to simultaneously trace the lineage of cells and reconstruct the evolutionary history of the mitochondria from single-cell sequencing data. Single-cell sequencing measures mutations from a mixture of the mitochondrial clones in each cell. MERLIN deconvolves this data to infer a concordant pair of a cell lineage tree, describing the ancestral relationships of the cells, and a mitochondrial clone tree, describing the evolution of the mitochondrial clones. Underpinning the concordance of these trees is the concept of “nested perfect phylogenies”, which models the joint process of mitochondrial evolution and inheritance of mitochondrial clones between cells during cell division. On simulated data, we show that MERLIN outperforms single-cell lineage tracing methods tailored for nuclear mutations. MERLIN also infers more accurate mitochondrial clone trees compared to existing methods for clone tree inference from bulk sequencing data that do not model the concordance of the clone tree with a cell lineage tree. On single-cell whole-genome sequencing data of a gastric cancer cell line, MERLIN infers more biologically plausible cell lineage tree and mitochondrial clone tree compared to existing methods.

There are several avenues for future research. First, the current problem formulation minimizes the *L*_1_ norm of the error between the measured mitochondrial mutation frequency matrix *F* and the factorization *BU*. Alternatively, one could incorporate a probabilistic model for the read count data under a maximum likelihood framework, similar to several bulk and single-cell tumor phylogeny methods (Singer *et al.* 2018, Malikic *et al.* 2019b, Sashittal *et al.* 2023). Second, MERLIN makes the infinite site assumption (Farris 1977) and assumes that all mitochondrial clones in the parent cell are inherited by the daughter cells during cell division. A statistical model that accounts for violations of the infinite sites assumption and uneven distribution of clones between the daughter cells during cell division may lead to improvement in the inference of the cell lineage tree. Third, several single-cell DNA sequencing technologies (Zhang *et al.* 1992, Klein *et al.* 1999, Arneson *et al.* 2008) simultaneously measure nuclear and mitochondrial mutations in individual cells. As such, the proposed framework of using mitochondrial mutations are phylogenetic markers for cell lineage tracing introduced in this paper can be expanded to include nuclear mutations, improving the resolution of the inferred cell lineage trees. Fourth, since mitochondrial reads are captured with single-cell RNA sequencing, mitochondrial mutations can be used to validate the cell lineage trees build using lineage tracing data from recently developed CRISPR-Cas9-based single-cell lineage tracing technologies (McKenna *et al.* 2016, Raj *et al.* 2018, Chan *et al.* 2019). Lastly, given the strong connection between mitochondrial lineage tracing and MSC, we anticipate that the theoretical and algorithmic advancements for solving the MSC problem can be translated to the problem of single-cell lineage tracing using mitochondrial mutations as phylogenetic markers (Mirarab *et al.* 2021). Lastly, given the strong connection between mitochondrial lineage tracing and MSC, we anticipate that the theoretical and algorithmic advancements for solving the MSC problem can be translated to the problem of single-cell lineage tracing using mitochondrial mutations as phylogenetic markers (Mirarab *et al.* 2021).

Mitochondrial mutations are excellent phylogenetic markers for lineage tracing because of their high mutation rate and compatibility with concurrent single-cell measurements of cell state. As such, recent years have witnessed rapid development of specialized single-cell and spatial sequencing technologies that employ mitochondrial transcriptome enrichment to enhance mitochondrial genome coverage (Lareau *et al.* 2021, Miller *et al.* 2022, Xue *et al.* 2023). We envision that MERLIN will play a crucial role in the analyses of these data and provide a foundation for future development of algorithms for mitochondrial lineage tracing.

## Supplementary Material

btae231_Supplementary_Data

## Data Availability

MERLIN software, synthetic datasets, processed MKN-45 scDNA-seq data, and code to reproduce the results figures are available at https://github.com/raphael-group/MERLIN. The MKN-45 single-cell DNA sequencing data are available in the SRA (accession number PRJNA498809) and in the Gene Expression Omnibus (GEO accession number GSE142750).
