# Supplementary material for: Joint inference of cell lineage and mitochondrial evolution from single-cell sequencing data
Source: Bioinformatics. 2024 Jun 28;40(Suppl 1):i218–27. doi: 10.1093/bioinformatics/btae231 (PMC11211840; doi:10.1093/bioinformatics/btae231)
Supplement: btae231_Supplementary_Data [file btae231_supplementary_data.zip › btae231_Supplementary_Data/ISMB_2024_Supplement_266.pdf]

Joint inference of cell lineage and mitochondrial evolution from single-cell sequencing data

Palash Sashittal[1], Viola Chen[1], Amey Pasarkar[1], and Benjamin Raphael[1,*]

[1]Dept. of Computer Science, Princeton University, NJ 08540
[*]Correspondence: braphael@princeton.edu

Abstract

Eukaryotic cells contain organelles called mitochondria that have their own genome. Most cells contain thousands of mitochondria which replicate, even in non-dividing cells, by means of a relatively error-prone process resulting in somatic mutations in their genome. Because of the higher mutation rate compared to the nuclear genome, mitochondrial mutations have been used to track cellular lineage, particularly using single-cell sequencing that measures mitochondrial mutations in individual cells. However, existing methods to infer the cell lineage tree from mitochondrial mutations do not model *heteroplasmy*, which is the presence of multiple mitochondrial clones with distinct sets of mutations in an individual cell. Single-cell sequencing data thus provides a mixture of the mitochondrial clones in individual cells, with the ancestral relationships between these clones described by a mitochondrial clone tree. While deconvolution of somatic mutations from a mixture of evolutionarily related genomes has been extensively studied in the context of bulk sequencing of cancer tumor samples, the problem of mitochondrial deconvolution has the additional constraint that the mitochondrial clone tree must be concordant with the cell lineage tree. We formalize the problem of inferring a concordant pair of a mitochondrial clone tree and a cell lineage tree from single-cell sequencing data as the NESTED PERFECT PHYLOGENY MIXTURE (NPPM) problem. We derive a combinatorial characterization of the solutions to the NPPM problem, and formulate an algorithm, MERLIN, to solve this problem exactly using a mixed integer linear program. We show on simulated data that MERLIN outperforms existing methods that do not model mitochondrial heteroplasmy nor the concordance between the mitochondrial clone tree and the cell lineage tree. We use MERLIN to analyze single-cell whole genome sequencing data of 5220 cells of a gastric cancer cell line and show that MERLIN infers a more biologically plausible cell lineage tree and mitochondrial clone tree compared to existing methods.

Code availability: Software is available at `https://github.com/raphael-group/MERLIN`

A Proofs

A.1 Proof for Theorem 2

Theorem 3.2. *A frequency matrix F admits a solution to the ENPPM problem if and only if (i) binarization F' of F is a perfect phylogeny matrix and (ii) there exists a spanning arborescence H of ancestry graph G of F such that, for all $i \in \{1, \ldots, n\}$, $f_{i,j} > \sum_{j' \in \delta^+_H(j)} f_{i,j'}$ if $f_{i,j} > 0$ and $\sum_{j' \in \delta^+_H(j)} f_{i,j'} = 0$ if $f_{i,j} = 0$.*

Proof. We first want to recall the lemma 4 from El-Kebir *et al.* [31],

Lemma A.1. *Given a frequency matrix F where $f_{i,j}$ denote the frequency of mutation j in cell i, and a clonal matrix B, for the unique solution to the problem $F = UB$, we have*

$$u_{i,j} = f_{i,j} - \sum_{j' \in \delta_S(j)} f_{i,j'}$$

We will use this lemma in our proof.

($\Rightarrow$) Let $F = UB$ be the ENPPM solution. By definition, we know that U and B are concordant. Let S be the clone tree corresponding to clone matrix B. We will show that both condition (i) and (ii) in Theorem 2 holds.

Condition (i) We must show that binarization F' of F is a perfect phylogeny matrix. Let σ be a function that maps each clone ℓ to the mutation j on the edge incoming to clone ℓ in S (since S is a tree, there is a unique incoming edge to each clone ℓ in S). Since $F = UB$, we have the following relationship between the frequencies $f_{i,\sigma(\ell)}$ and proportions U of the clones,

$$f_{i,\sigma(\ell)} = u_{i,\ell} + \sum_{\ell' : \ell \preceq_S \ell'} u_{i,\ell'}.$$

Clearly, if $u_{i,\ell} > 0$, then $f_{i,\sigma(\ell)} > 0$. On the other hand, if $u_{i,\ell} = 0$ then since U and B are concordant, we have $u_{i,\ell'} = 0$ if $\ell \preceq_S \ell'$. This is because, if $u_{i,\ell} = 0$ and $u_{i,\ell'} > 0$, then $\ell \not\preceq_T \ell'$ where T is the cell lineage tree corresponding to U, which contradicts the premise that U and B are concordant. As such, $f_{i,\sigma(\ell)} = 0$ if $u_{i,\ell} = 0$. Therefore, in the binarization F' of F, $f'_{i,\sigma(\ell)} = 1$ if and only if $u'_{i,j} = 1$. Since U is from a solution of the ENPPM problem, U' is a perfect phylogeny. Therefore, F' which is made of a permutation of columns of U' is also a perfect phylogeny.

Condition (ii) Clearly S is a spanning arborescence of the ancestry graph H. Since ENPPM is a constrained version of the PPM problem, UB is also a solution to the PPM problem. Therefore, the sum condition must hold (Theorem 3.1). We must show that for all cells i and mutation j, if $f_{i,j} > 0$, then $f_{i,j} > \sum_{j' \in \delta_S(j)} f_{i,j'}$. We show this by contradiction. Let there be cell i and mutation j such that $f_{i,j} \leq \sum_{j' \in \delta_S(j)} f_{i,j'}$ and $f_{i,j} > 0$. Since the sum condition still holds, we must have $f_{i,j} = \sum_{j' \in \delta_S(j)} f_{i,j'}$, which implies that $u_{i,\ell} = 0$ where $\sigma(\ell) = j$. Since $f_{i,j} > 0$ and $u_{i,\ell} = 0$, there must be at least one mutation $j' \succ_S j$ such that $u_{i,\ell'} > 0$, where $\sigma(\ell') = j'$. However, since $u_{i,\ell} = 0$ and $u_{i,\ell'} > 0$, ℓ can not precede ℓ' in the cell lineage tree T induced by the binarization U' of U, i.e. $\ell' \not\succ_T \ell$. However, this together with $j' \succ j$ means that the partial order on the mitochondrial clones induced by cell lineage tree T is not an extension of the partial order induced by the clone tree S, which contradicts the premise that T and S are concordant.

($\Leftarrow$) Consider the clone tree S corresponding to the spanning arborescence H of ancestry graph G such that condition (ii) is satisfied. Note that condition (ii) is an restriction of the sum condition described in Theorem

1. Thus, if Condition (ii) is true, ie. $f_{i,j} > \sum_{j' \in \delta_S(j)} f_{i,j'}$ then it also satisfies the condition in Theorem 1, ie. $f_{i,j} \geq \sum_{j' \in \delta_S(j)} f_{i,j'}$. Let $F = UB$, where B is the clone matrix corresponding to the clone tree S. Since B is a perfect phylogeny matrix, without loss of generality, we assume that the clone matrix B is lower triangular with $b_{j,j} = 1$ for each mutation j [31]. We need to show that the binarization U' of U admits a perfect phylogeny and that U is concordant with B.

We start by showing that binarization U' of U is a perfect phylogeny. By lemma A.1, we know that if $f_{i,j} = 0$ then $u_{i,j} = 0$ as well. If $f_{i,j} > 0$, then by the Condition (ii), we get

$$f_{i,j} > \sum_{j' \in \delta_S(j)} f_{i,j'},$$

and therefore $u_{i,j} > 0$. As such, U' is identical to F' and since F' is a perfect phylogeny matrix, so is U'.

Now, we show that U and B are concordant by contradiction. Let us assume there exists a pair of clones ℓ and ℓ' such that $\ell \preceq_S \ell'$ and $\ell \not\preceq_T \ell'$. Since $\ell \not\preceq_T \ell'$, there must be at least one cell i such that $u_{i,\ell'} > 0$ and $u_{i,\ell} = 0$. Let σ be a function that maps each clone ℓ to the mutation j on the edge incoming to clone ℓ in clone tree S. Since $F = UB$, we have $f_{i,\sigma(\ell)} = u_{i,\ell} + \sum_{\ell' : \ell \preceq_S \ell'} u_{i,\ell'} > 0$. Since $f_{i,\sigma(\ell)} > 0$, by condition (i),

$$f_{i,\sigma(\ell)} > \sum_{j' \in \delta_S^+(\sigma(\ell))} f_{i,j'}.$$

From Lemma A.1, this implies that $u_{i,\ell} = f_{i,\sigma(\ell)} - \sum_{j' \in \delta_S^+(\sigma(\ell))} f_{i,j'} > 0$, which contradicts premise that $u_{i,\ell} = 0$. □

A.2 Proof for Theorem 3.3

Here, we provide to proof for theorem 3.3 by adapting the hardness proof for the PPM problem 2.1 provided in El-Kebir *et al.* [31]. For completeness, we restate the theorem.

Theorem 3.3. *The ENPPM problem is NP-complete, even for* $n = 2$.

Proof. Given a mixture matrix U and clone matrix B, it is easy to check if $F = UB$ and if U and B are evolutionarily concordant in polynomial time. Specifically, for the concordance, building a perfect phylogeny from a binary matrix, if it exists, and checking if a partial order is an extension of another, both can be done in polynomial time. As such, this problem is in NP.

We show that it NP-hardness via a reduction from the Subset Sum problem, which is known to be NP-complete [47]. In the Subset Sum Problem, we are given a a set of non-negative integers $A = \{a_1, \ldots, a_q\}$ and integer d, we are asked there exists a subset $A' \subset A$ such that the sum of the integers in A' is equal to d.

For a given instance (A, d) of the Subset Sum problem where A has q elements in increasing order (i.e. $a_p \leq a_{p+1}$), we construct frequency matrix F with $n = 2$ cells and $m = q + 2$ for the EMMP problem as follows,

$$F = \frac{1}{e} \begin{pmatrix} d & e-d & a_1 - \epsilon & a_2 - \epsilon & \ldots & a_q - \epsilon \\ e-d & d & q\epsilon & (q-1)\epsilon & \ldots & \epsilon \end{pmatrix}$$

where $e = \sum_{p=1}^{q} a_p$ and $0 < \epsilon < 1/(q+1)$. Clearly this construction takes polynomial time. The ancestry graph G corresponding to F is a directed acyclic graph with vertex 0 with outgoing edges to all other vertices $\{1, \ldots, q+2\}$. Additionally, vertex 1 has an outgoing edge to vertex $p+2$ if element $a_p < d$ for each $p \in \{1, \ldots, q\}$. Similarly, vertex 2 has an outgoing edge to vertex $p+2$ if element $a_p < e - d$ for

each $p \in \{1, \ldots, q\}$. We show that F admits a solution to the EMMP problem if and only if instance (A, d) admits a solution to the Subset Sum Problem.

($\Rightarrow$) Let H be a spanning arborescence of ancestry graph G that satisfies the conditions given in Theorem 3.2. Note that $\delta_H(0) = \{1, 2\}$ and because $f_{1,1} + f_{1,2} = 1$ and $f_{2,1} + f_{2,2} = 1$, H does not contain edges from vertex 0 to any vertex $p + 2$ for $p \in \{1, \ldots, q\}$. Let x be the sum of the frequencies of the children of vertex 1 in cell 1, i.e. $x = \sum_{p \in \delta_H(1)} f_{1,p}$. Since the total sum of the vertices $3, \ldots, q + 2$ is $1 - q\epsilon/e$, the sum of the frequencies of the children of vertex 2 in cell 1 will be $1 - x - q\epsilon/e$. We show the children of vertex 1 define the subset A' of A such that the sum of the elements is d. Specifically, since A is composed of integers, the x must be $(k - |A'|\epsilon)/e$, where k is an integer. We will show that k must be equal to d.

From the sum condition on vertex 1 we have that $x < d/e$ and from vertex 2 we have $x > (d - q\epsilon)/e$. if k is the sum of the elements of A', we have $x < k/e$ which together with constraint $x > (d - q\epsilon)/e$ implies that $k \geq d$ since $q\epsilon < 1$. Similarly, since $|A'| < q$, we have $x > (k - q\epsilon)/e$ which together with $x < d/e$ implies that $k \leq d$. Putting these together we get $k = d$ which concludes the forward direction of the proof.

$\Leftarrow$ Let A' be the set whose sum equals d and thus the sum of $A \setminus A'$ is $e - d$. Consider the spanning arborescence H of G composed of the following set of edges: $\{(0, 1), (0, 2)\} \cup \{(1, p + 2) : a_p \in A', p \in \{1, \ldots, q\}\} \cup \{(2, p + 2) : a_p \notin A', p \in \{1, \ldots, q\}\}$. It is easy to check that this spanning arborescence satisfies the sum condition and the since the binarization F' of the frequency matrix F admits a perfect phylogeny, this concludes the proof.

□

B Algorithmic details

B.1 Ancestry graph

In practice frequency matrix has errors and, as such, there may not be an exact factorization UB of the frequency matrix F such that U and B are evolutionarily concordant. As such, we build an approximate ancestry graph G in two steps. First, we first build a correlation graph C to cluster mutations are that co-occurring with similar frequencies across cells. Correlation graph is an undirected graph with vertices representing the mutations $1, \ldots, m$, and it contains edge (j, j') if the frequencies of mutations j and j' are more correlated than a user-defined threshold. We cluster mutations are finding the maximum clique cover of the correlation graph C, where each clique corresponds to a cluster of mutations. For each cluster, we compute the average frequency across the mutations in the cluster for each cell. Second, we now build the ancestry graph G on the clustered mutations. Suppose there are k clusters.

B.2 Perfect phylogeny constraints

We require that the binarization U' of the mixture matrix U is a perfect phylogeny. We use the set inclusion and disjointness (SID) formulation described in Chimani *et al.* [48]. This formulation uses a characterization of perfect phylogeny matrices that states that for any two columns, we require the 1-sets, i.e. set of rows i such that $u'_{i,j} = 1$, of any two columns should either be disjoint or related by containment [29]. We introduce continuous variables $y_{j,j'}$ and $z_{j,j'}$ for each pair of mutations j and j'. We force $y_{j,j'} = 0$ if the 1-set of mutation j is not contained in 1-set of mutation j' using the following constraint for each cell i,

$$y_{j,j'} \leq 1 - u'_{i,j} + u'_{i,j'}.$$

Along the same vein, we enforce $z_{j,j'} = 0$ if the 1-set of mutation j is not disjoint with 1-set of mutation j' using the following constraint for each cell i,

$$z_{j,j'} \leq 2 - u'_{i,j} - u_{i,j'}.$$

For U' to be a perfect phylogeny matrix, we require that for any two mutations j and j', the 1-sets should be either related by containment or be disjoint. In other words, at least one of $y_{j,j'}$, $y_{j',j}$ and $z_{j,j'}$ must be 1 for each pair of mutation j and j'. We achieve this by enforcing the following constraint for each pair j, j' of mutations,

$$y_{j,j'} + y_{j',j} + z_{j,j'} \geq 1.$$

In summary, to enforce that U' is a perfect phylogeny matrix we introduce $O(m^2)$ continuous variables and $O(nm^2)$ constraints.

B.3 Complete MILP

For completeness sake, we describe the complete MILP here.

$$\begin{aligned}
\min \quad & \sum_{i=1}^{n}\sum_{j=1}^{m} c_{i,j} & \\
\text{s.t.} \quad & \sum_{e \in \delta^-_G(j)} x_e = 1, & \forall j \in [m], \\
& u_{i,\ell} \leq u'_{i,\ell} & \forall i \in [n], \ell \in [m], \\
& u_{i,\ell} \geq \mu u'_{i,\ell} & \forall i \in [n], \ell \in [m], \\
& u'_{i,\ell} \geq x_e + u'_{i,\ell'} - 1, & \forall e = (\ell, \ell') \in E(G), i \in [n], \\
& y_{j,j'} \leq 1 - u'_{i,j} + u'_{i,j'}, & \forall j \in [m], j' \in [m], j \neq j', \\
& z_{j,j'} \leq 2 - u'_{i,j} - u_{i,j'}, & \forall j \in [m], j' \in [m], j < j', \\
& y_{j,j'} + y_{j',j} + z_{j,j'} \geq 1, & \forall j \in [m], j' \in [m], j < j', \\
& \hat{f}_{i,j} = u_{i,j} + \sum_{(j,j') \in \delta^+_S(j)} \hat{f}_{i,j'}, & \forall i \in [n], j \in [m], \\
& h_{i,e} \leq \hat{f}_{i,j'}, & \forall i \in [n], e = (j, j') \in E(G), \\
& h_{i,e} \leq x_e, & \forall i \in [n], e = (j, j') \in E(G), \\
& h_{i,e} \geq \hat{f}_{i,j'} + x_e - 1., & \forall i \in [n], e = (j, j') \in E(G), \\
& \hat{f}_{i,j} = u_{i,j} + \sum_{e \in \delta^+_G(j)} h_{i,e}, & \forall i \in [n], j \in [m], \\
& c_{i,j} \geq f_{i,j} - \hat{f}_{i,j}, & \forall i \in [n], j \in [m], \\
& c_{i,j} \geq \hat{f}_{i,j} - f_{i,j}, & \forall i \in [n], j \in [m], \\
& x_e \in \{0, 1\}, & \forall e \in E(G) \\
& u'_{i,\ell} \in \{0, 1\}, & \forall i \in [n], \ell \in [m] \\
& u_{i,\ell} \in [0, 1], & \forall i \in [n], \ell \in [m] \\
& h_{i,e} \in [0, 1], & \forall e \in E(G), i \in [n] \\
& \hat{f}_{i,j} \in [0, 1], & \forall i \in [n], j \in [m] \\
& c_{i,j} \in [0, 1], & \forall i \in [n], j \in [m] \\
& y_{j,j'} \in [0, 1], & \forall j \in [m], j' \in [m], j \neq j' \\
& z_{j,j'} \in [0, 1], & \forall j \in [m], j' \in [m], j < j'.
\end{aligned}$$

C Simulation details

We generated simulated data with $n = 50, 100, 500$ cells and $m = 5, 10, 15$ mitochondrial mutations, and 5 instances for each set of parameters. For each simulation instance, we obtain the ground truth clone tree, mutation to clone assignment, cell lineage tree, and cell-to-clone assignments, which are used in assessment of performance of various methods. In the following, we describe our procedure to simulate the mitochondrial clone tree and cell linage tree under the nested phylogeny model.

C.1 Mitochondrial Clone Tree Generation

We used a growing random network [33] to generate a clone tree representing the ancestral relationships among mitochondrial clones. By the perfect phylogeny model, two vertices connected by an edge differs by one and exactly one mutation, and that no mutation arises twice.

C.2 Cell Lineage Tree Generation

With the assumption that cell lineage tree is also a perfect phylogeny, we assumed that the cell lineage trees can be represented by a tree with m edges, where each edge in the tree represents the appearance of 1 mutation. Since each mutation appears exactly once, we will have exactly m edges in the tree, producing a tree with $m + 1$ vertices, with the root of the tree having no mutations. As such, the cells are assumed to be clustered into $m + 1$ clusters.

To ensure the concordance of cell lineage tree and clone tree, for each clone, we allow it to be assigned to a cluster if and only if its parent in clone tree is present in the cluster. In code, this translates to only allow a clone to be added to the subtree rooted at its parent in clone tree.

```
for clone in clones:
    if clone == 0: continue # root of tree
    parent_in_clone_tree = clone_tree[clone].parent
    subtree = get_subtree(cell_tree, parent_in_clone_tree)
    parent_in_cell_tree = random.choice(tuple(subtree))
    cell_tree.add_edge(parent_in_cell_tree, clone)
```

C.3 Simulation of Usage Matrix and Read Counts

Allele frequencies are sampled from a Dirichlet distribution. We re-sample the proportions of mitochondrial clones until all the mitochondrial clones present in the cell have proportion greater than $\mu = 0.05$. Read counts are then simulated from a beta-binomial distribution with probability of observed a variant read given by the allele frequency $F = UB$ of the mutations and precision parameter $s = 15$.

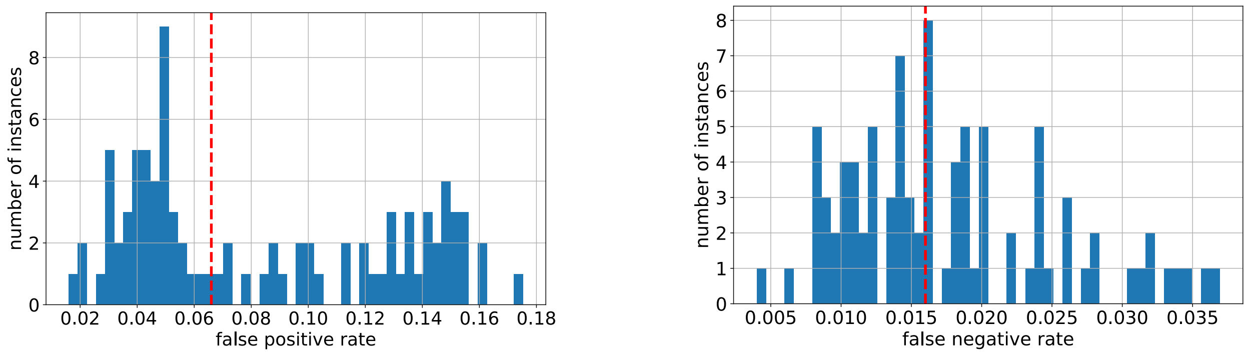

(a) *(b)*

Fig. S1 Histogram of the (a) false positive rate α and the (b) false negative rate β in the observed mutation matrix A' of simulated instances. Red lines show the median false positive rate (0.066) and median false negative rate (0.016).

	command-line keyword	description
n	${num_cells}$	number of cells
m	${num_mutations}$	number of mutations
Q	${input_variant_readcounts}	variant allele read count matrix
R	${input_total_readcounts}	total read count matrix

Table S1 Description of the keywords used in the command-line arguments.

D Method Parameters

Here, we provide the precise commands used to run competing methods for benchmarking on the simulated data. We use the median false positive rate $\alpha = 0.066$ and false negative rate $\beta = 0.016$ in the observed mutation matrix generated from the simulated data (Figure S1).

We note that not all competing methods take the same inputs. Pairtree takes total read counts and variant read counts in a custom format (.ssm), SCITE, PhISCS and PhISCS-BnB take a binary mutation matrix with potential missing values, and CALDER takes a matrix with alternating columns being total and variant read counts. To generate input to those methods, we used custom python scripts to modify the input format. For those taking a binary input, we used the VAF threshold of 0.05 as the cutoff for binarization. The detailed preparation scripts are published on Github.

Description of keywords used in the command-line arguments are provided in Table S1.

MERLIN

```
python merlin.py
-t ${input_total_readcounts} -v ${input_variant_readcounts}
-o ${output_prefix}
```

Pairtree

```
pairtree --params ${input_json} {input.ssm} ${output_prefix}$
```

SCITE We run SCITE for 1000000 iterations. Since we do not have any homozygous mutations in the simulations, we set -cc flag to 0.

```
scite -i ${input_character_matrix} -n ${num_mutations}$ -m ${num_cells}$
-o ${output_prefix}$ -a -cc 0 -l 1000000 -r 1
-fd 0.066 -fn 0.016 0 -max_treelist_size 1
```

PhISCS We used a Python script to generate the input matrix to PhISCS and PhISCS-BnB. We compute the variant allele frequency (VAF) and consider a mutation to be present if it has VAF > 0.05.

```
python PhISCS-B --SCFile ${single_cell_matrix}$
-time 3600 -fn 0.016 -fp 0.066 -o ${output_prefix}$
```

PhISCS-BnB

```
python ${PhiSCS-BnB_directory}$/main.py
-i ${single_cell_matrix}$
-o ${output_prefix}$
```

CALDER We used a Python script to generate the required character matrix input from the simulation output - total read count and variant read count. The read count of reference and alternative allele is computed and put into neighboring columns of the matrix.

```
java -jar calder.jar -i ${input_character_matrix}$
-o ${output_directory}$
```

E Performance of MERLIN in the presence of mutation loss

MERLIN makes assumptions about both clonal tree and cell lineage tree being perfect phylogenies, which imply that mutations are never lost, and all daughter clones will inherit all mutations from parental clone.

Realizing that these assumptions may not hold for all real-world scenarios, we simulated instances in which mutations are lost or not inherited with 5% probability in all cells. MERLIN's performance in the case of mutation loss demonstrates that MERLIN still performs well when the assumptions are violated in some small proportion of cells. Thus, MERLIN is robust against minor violations to model assumptions.

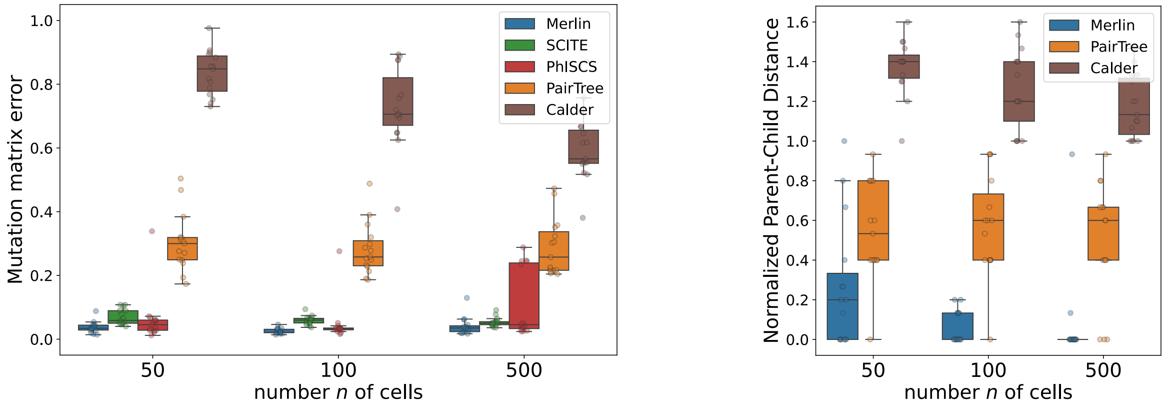

(a) *(b)*

Fig. S2 **MERLIN outperforms existing methods in reconstructing the mitochondrial clone tree and the cell lineage tree on simulations that include loss of mitochondrial mutations.** (a) Mutation matrix error and (b) normalized parent-child distance for each method on the simulated instances with mutation losses. Box plots show the median and the interquartile range (IQR), and the whiskers denote the lowest and highest values within 1.5 times the IQR from the first and third quartiles, respectively.

F Measure of Discordance

Here, we provides details of how we measure the discordance in Pairtree results. We pose the following problem to remove cells so that the results become concordant.

Problem F.1. *Given a mixture matrix U and clone matrix B, remove fewest rows of U to obtain a mixture matrix $\overline{U}$ that is concordant with the clone matrix B.*

The measure of discordance is number of cells that must be removed to make the mixture matrix concordant with the mitochondrial clone matrix. If U and B are already concordant, this measure is 0. It is easy to show that Problem F.1 is NP-hard by reduction from the Row Deletion Problem [49]. We formulate the following ILP to select the maximum number of rows from the binarization U' of the mixture matrix U to make the results concordant.

$$
\begin{aligned}
\max \ & \sum_{i=1}^{n} x_i \\
\text{s.t.} \ & \bar{u}_{i,j} \leq x_i, && \forall i \in [n], j \in [m], \\
& \bar{u}_{i,j} \leq u'_{i,j}, && \forall i \in [n], j \in [m], \\
& \bar{u}_{i,j} \geq x_i + u'_{i,j} - 1, && \forall i \in [n], j \in [m], \\
& y_{j,j'} \leq 1 - u'_{i,j} + u'_{i,j'}, && \forall j \in [m], j' \in [m], j \neq j', \\
& z_{j,j'} \leq 2 - u'_{i,j} - u_{i,j'}, && \forall j \in [m], j' \in [m], j < j', \\
& y_{j,j'} + y_{j',j} + z_{j,j'} \geq 1, && \forall j \in [m], j' \in [m], j < j', \\
& x_i \in \{0,1\}, && \forall i \in [n] \\
& \bar{u}_{i,j} \in [0,1], && \forall i \in [n], j \in [m] \\
& y_{j,j'} \in [0,1], && \forall j \in [m], j' \in [m], j \neq j' \\
& z_{j,j'} \in [0,1], && \forall j \in [m], j' \in [m], j < j'.
\end{aligned}
$$

$x_i = 1$ indicates that row i of U is selected and when $x_i = 0$, it means that row i is removed. The measure of discordance is the fraction of cells that are removed, i.e. fraction of rows that have $x_i = 0$. Formally it is given by $1 - \sum_{i=1}^{n} x_i/n$.
