# Supplementary figures and images for: Joint inference of cell lineage and mitochondrial evolution from single-cell sequencing data
Source: Bioinformatics. 2024 Jun 28;40(Suppl 1):i218–27. doi: 10.1093/bioinformatics/btae231 (PMC11211840; doi:10.1093/bioinformatics/btae231)

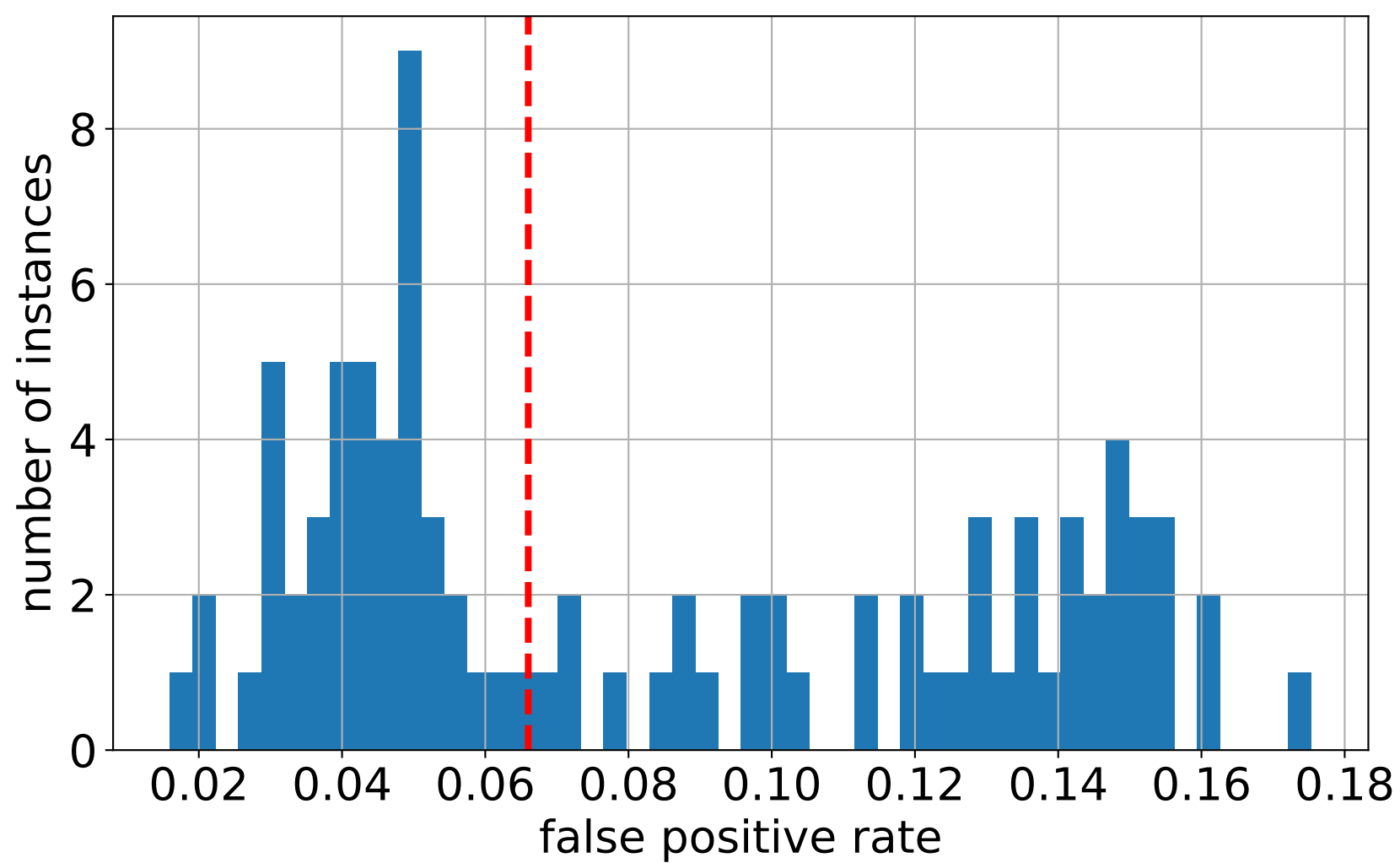

Supplement: btae231_Supplementary_Data [file btae231_supplementary_data.zip › btae231_Supplementary_Data/Raphael.266.supp.fig.1a.pdf]

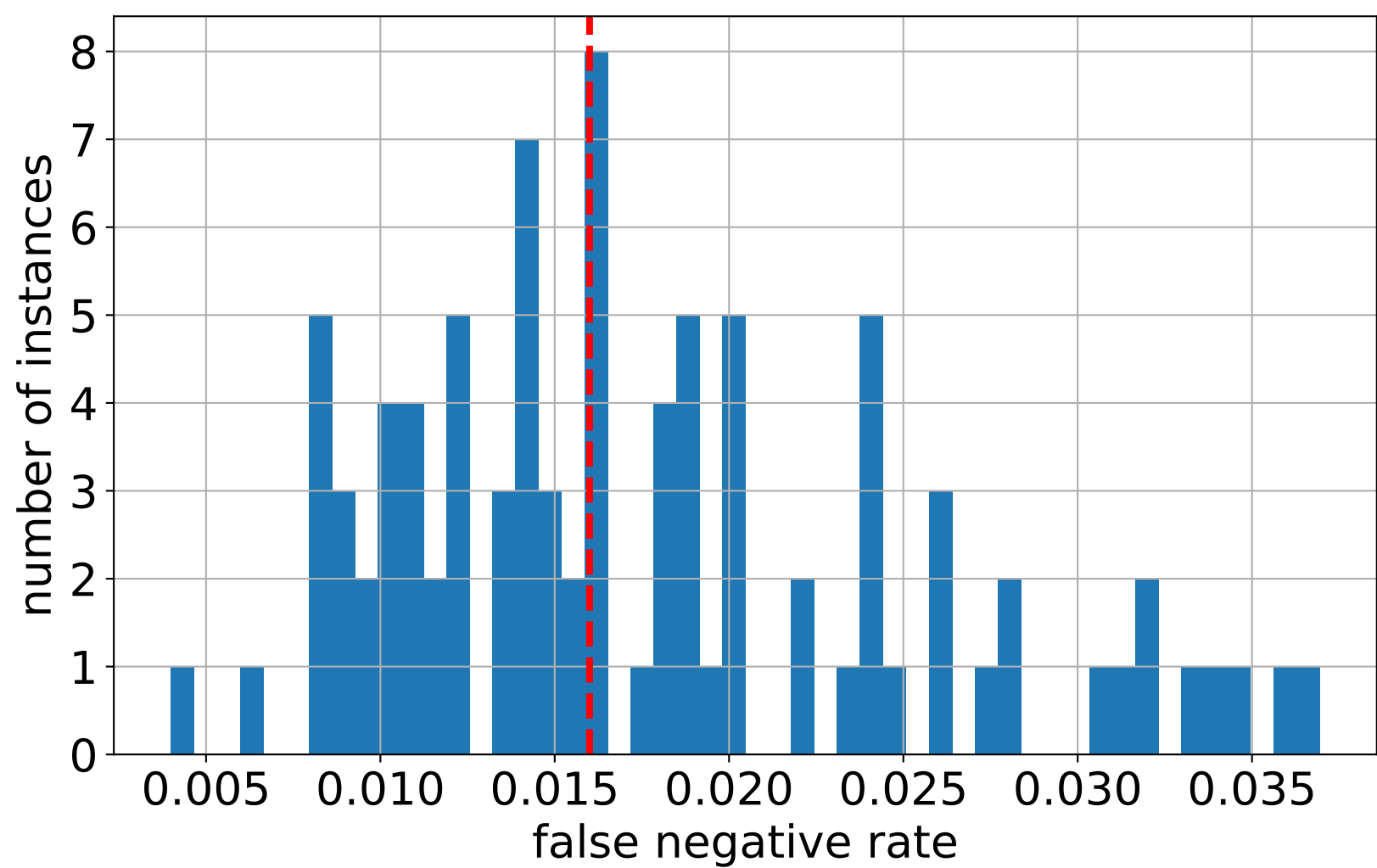

Supplement: btae231_Supplementary_Data [file btae231_supplementary_data.zip › btae231_Supplementary_Data/Raphael.266.supp.fig.1b.pdf]

Mutation matrix error

1.0  
0.8  
0.6  
0.4  
0.2  
0.0

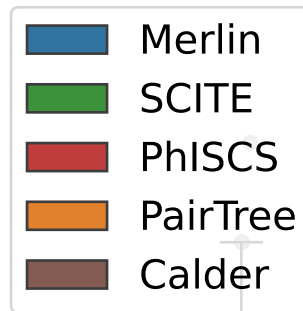

50

100

500

number  $n$  of cells

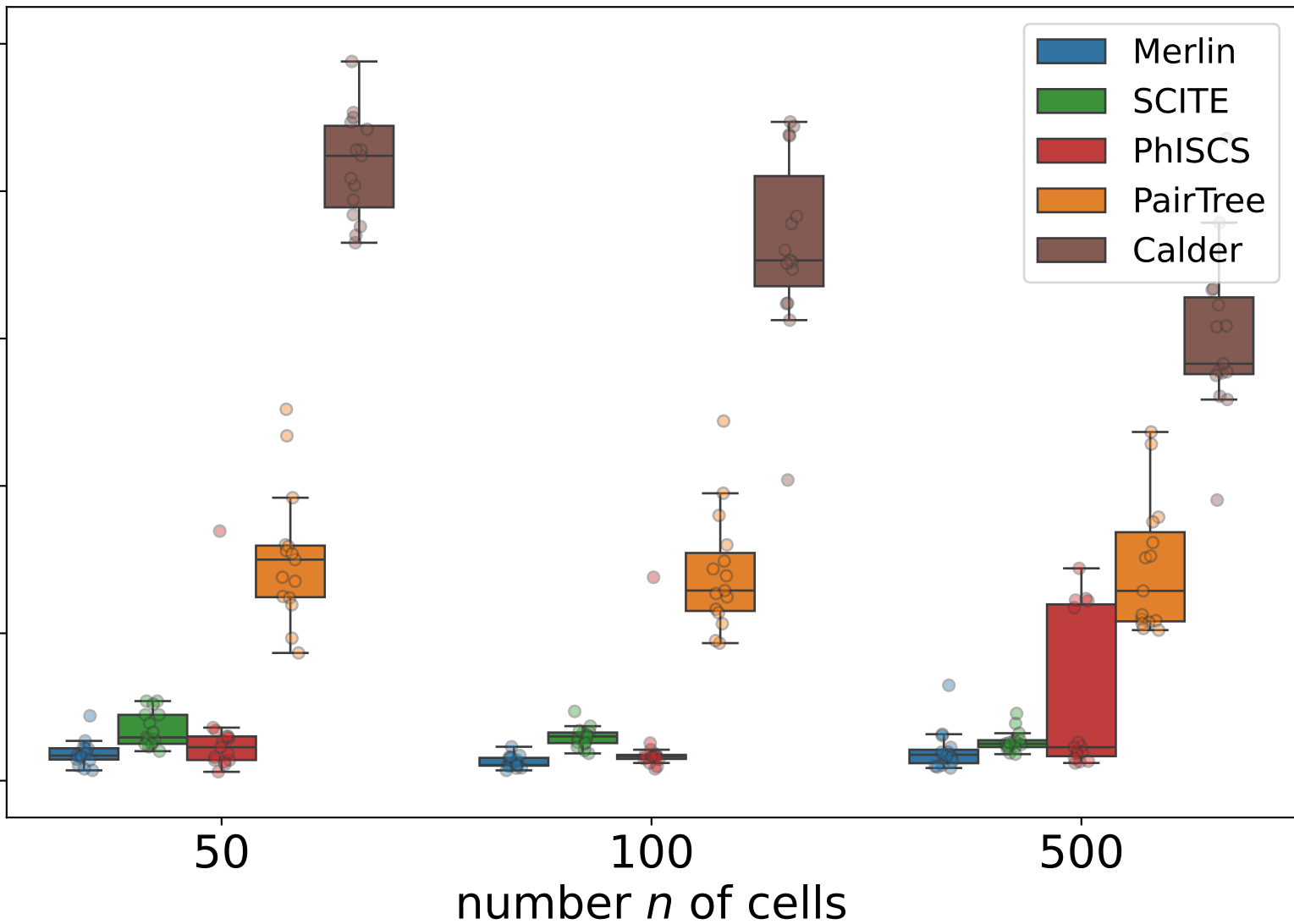

Supplement: btae231_Supplementary_Data [file btae231_supplementary_data.zip › btae231_Supplementary_Data/Raphael.266.supp.fig.2a.pdf]

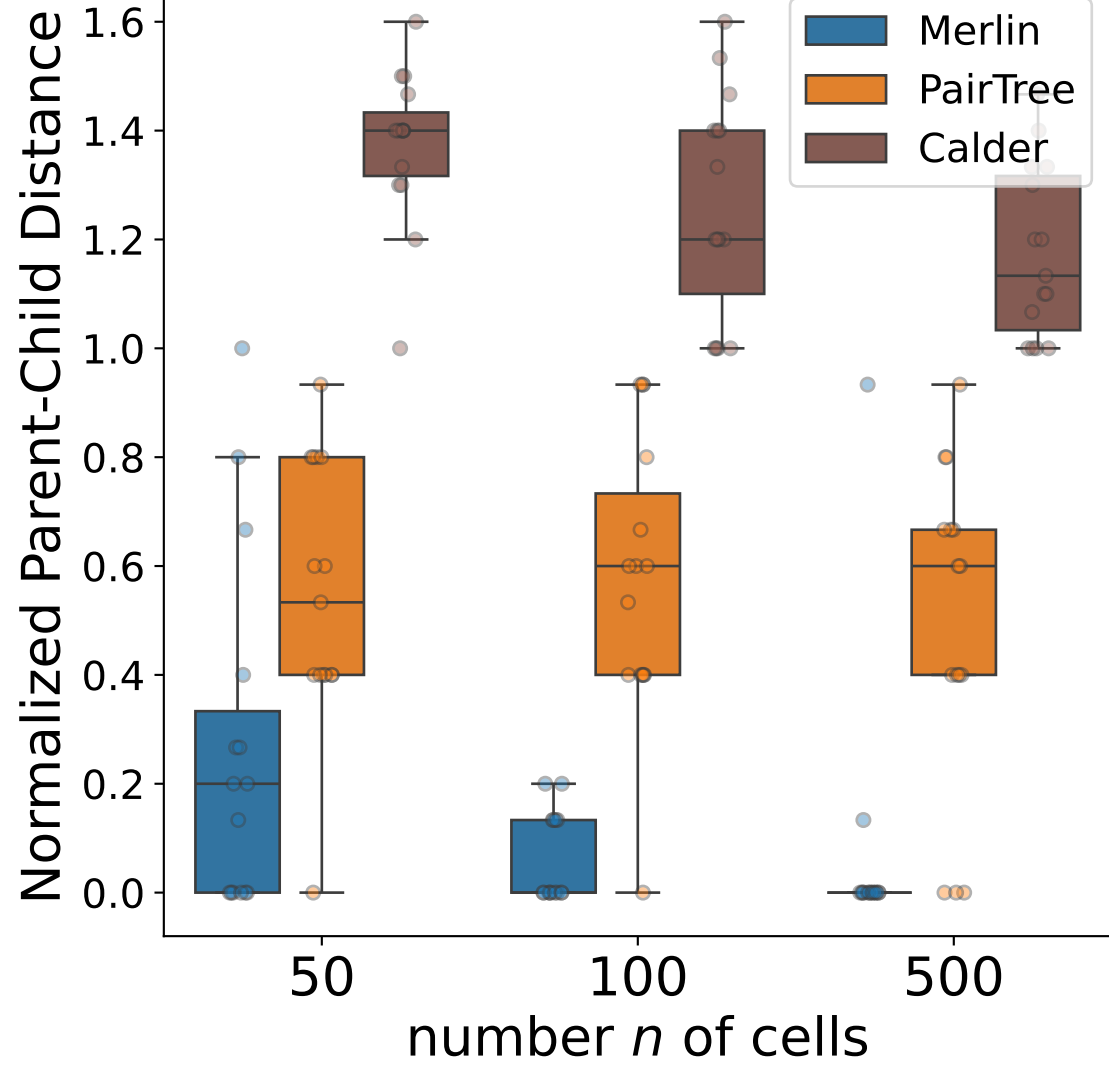

Supplement: btae231_Supplementary_Data [file btae231_supplementary_data.zip › btae231_Supplementary_Data/Raphael.266.supp.fig.2b.pdf]
